# Supplementary material for: Electromagnetic fields alter the motility of metastatic breast cancer cells
Source: Commun Biol. 2019 Aug 8;2:303. doi: 10.1038/s42003-019-0550-z (PMC6687738; doi:10.1038/s42003-019-0550-z)
Supplement: Supplementary file 1 — Supplementary Information [file 42003_2019_550_MOESM1_ESM.docx]

**SUPPLEMENTARY INFORMATION**

**Supplementary Figures**


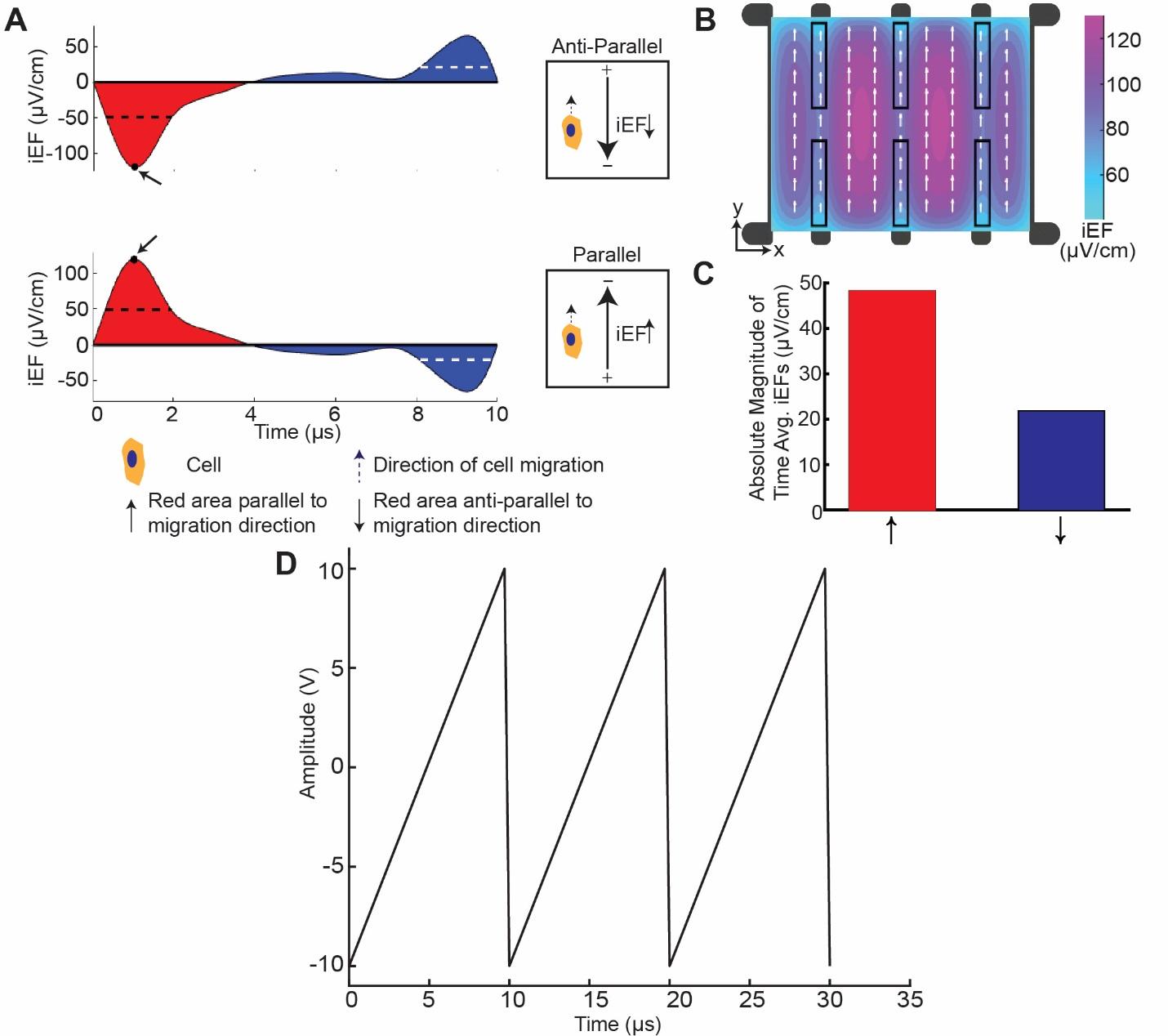


**Supplementary Figure 1. Characterization of the induced electric fields. (A) Top,** Red peak is aligned anti-parallel (↓) to the direction of cell migration. **Bottom,** iEFs applied parallel (↑) to the direction of migration as the red peak is in the same direction as cell migration. iEFs are applied at a frequency of 100 kHz and the graph is the variation of field for one time cycle (10 µs). **(B)** Variation of iEFs at its peak value (the arrow-head in (A)). In the viewing window, peak strength is between ~60-80 µVcm^-1^. **(C)** Time-averaged asymmetry in iEF for one time cycle (10 µs) from (A, Bottom) showing the average magnitude iEFs in parallel (↑) and anti-parallel (↓) directions. **(D)** Input sawtooth waveform to generate the asymmetric iEFs in (A), (B), and (C).


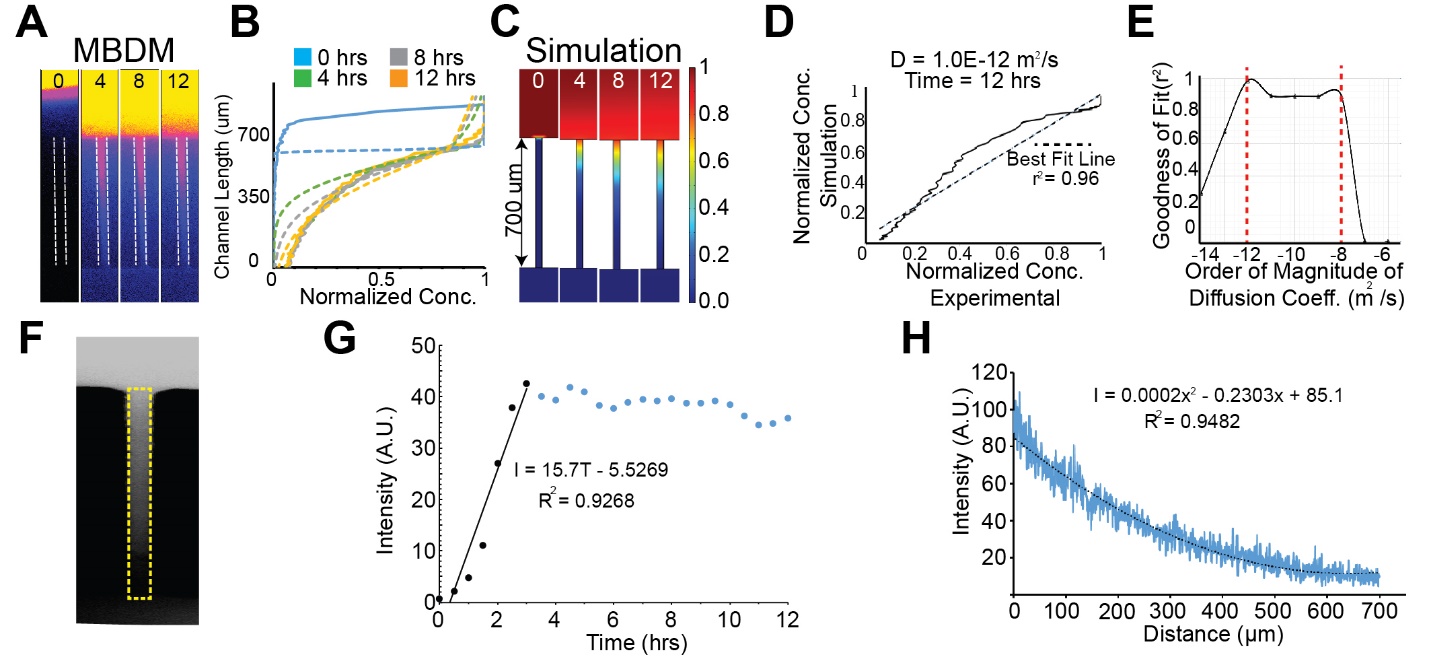


**Supplementary Figure 2. Chemokine gradient characterization. (A)** Fluorescent intensity map from experimental images such as that shown in (D) using 10 kDa FITC conjugated dextran dye. **(B)** Comparison of experimental (solid lines) to mathematical model (dotted lines) shows that they match each other closely. **(C)** Mathematical model of gradients in the MBDM assay plotted using COMSOL Multiphysics 5.2a. **(D)** Comparison of numerical model to the experimental at 12 hours for a diffusion coefficient of 1x10^-12^ m^2^s^-1^. **(E)** Predicted range of diffusion coefficient by the numerical model. **(F)** Representative region of interest in one microtrack used for experimental calculations. **(G)** Change in average intensity of the dye over time in the microtracks. **(H)** Normalized fluorescent intensity of dye over the length of the microtrack at the 12-hour time-point indicating a stable chemokine gradient in the MBDM assay.


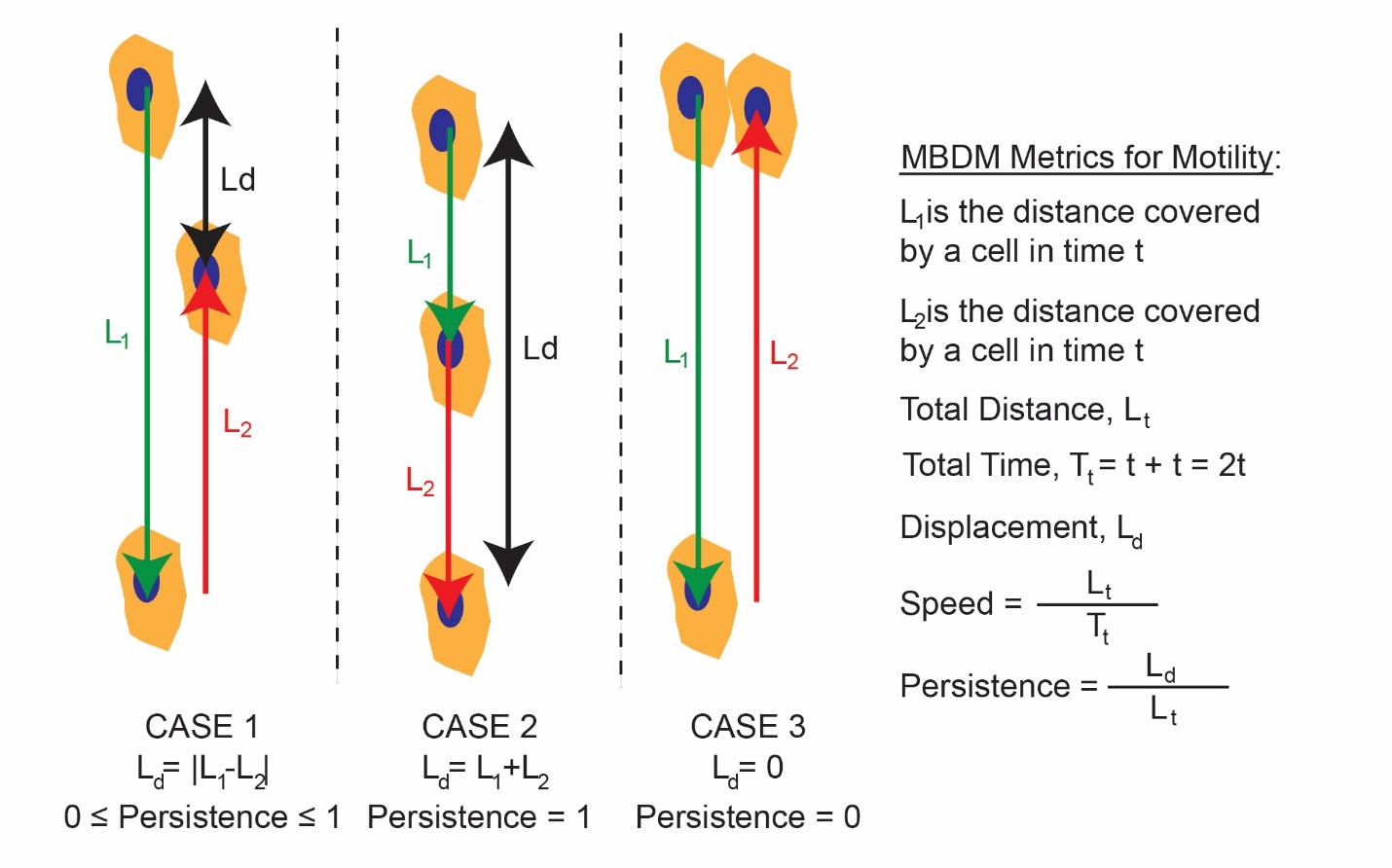


**Supplementary Figure 3. Quantitative metrics for cell motility enabled by MBDM Assay.** The cell migrates a distance L_1_ in time t and then a distance L_2_ in the same time t. Therefore, the total distance travelled by cell during tracking is L_1_+L_2_ in time 2t. Thus, mean speed is defined as the ratio of the total distance (in this case L_1_+L_2_) to the total time taken (2t). Persistence is defined as the ability of a cell to maintain a single direction of motion. Mathematically, it is the ratio of the cell displacement to the total distance (L_1_+L_2_) travelled by the cell. Displacement is defined as the shortest distance (L_d_) from the initial position to the final position of the cell. If the cell maintains a unidirectional motion then total distance is equal to displacement and therefore persistence equals one, whereas if the cell ends up at the same point from where it started then displacement is zero and hence persistence is zero.

**
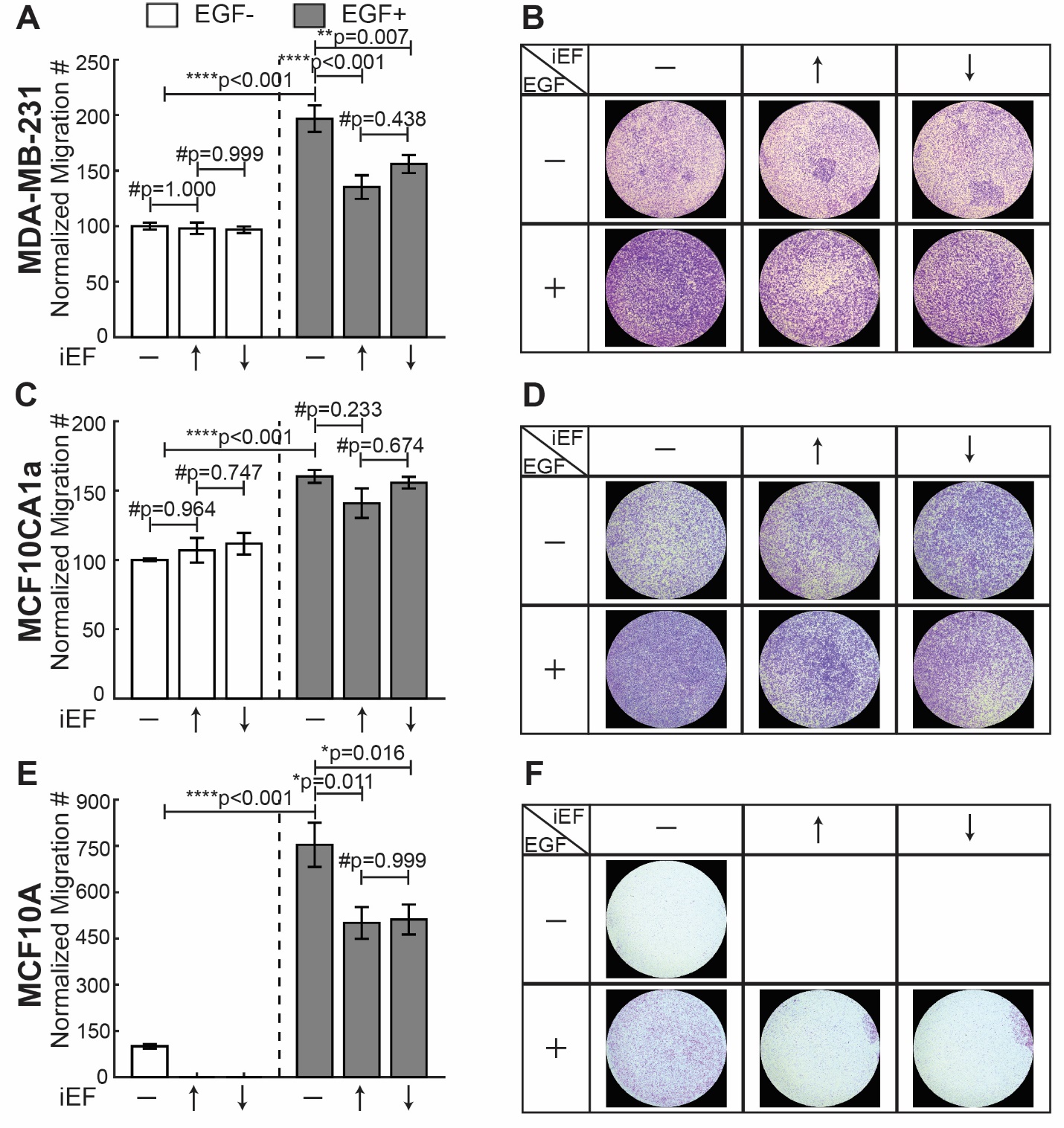
**

**Supplementary Figure 4. Modified transwell assay results. MDA-MB-231: (A)** iEF treatment had no effect on the total migration numbers when cells migrated in absence of exogenous EGF. However, iEFs bi-directionally reduced total migration numbers of cells migrating under EGF gradients. **(B)** Representative images of the wells for all the conditions for the data represented in (A). **MCF10CA1a: (C)** Parallel iEFs only reduce migration numbers in presence of EGF. **(D)**  Representative images of the wells for all the conditions for the data represented in (C). **MCF10A: (E)** iEFs bi-directionally significantly reduce migration numbers for cells migrating with EGF. **(F)** Representative images of the wells for all the conditions for the data represented in (E). All data presented as mean ± SE*_M_* (Unpaired two-tailed Student t-test, N = 3 for each condition).


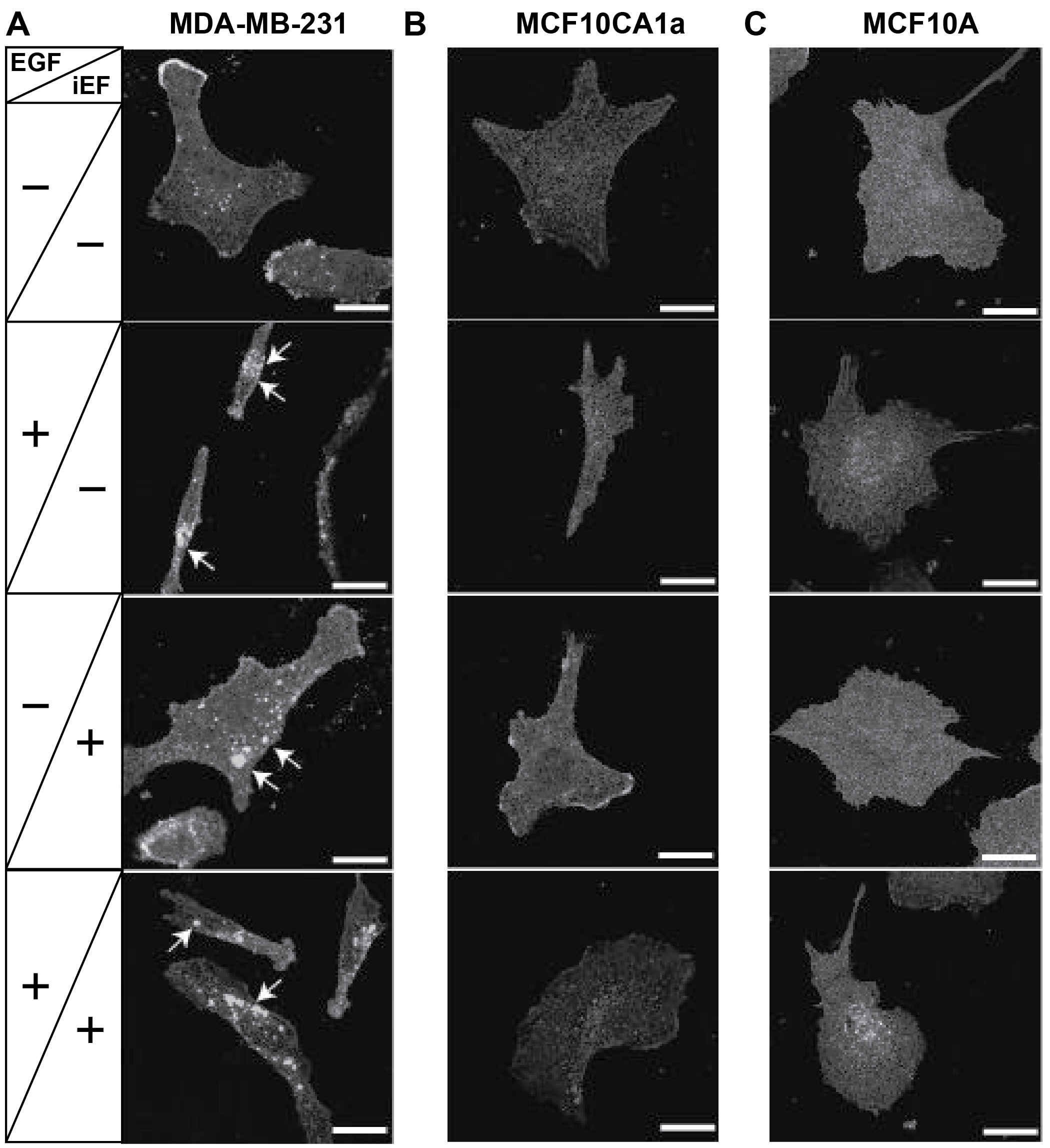


**Supplementary Figure 5. Effect of iEFs on EGFR distribution. (A) MDA-MB-231:** iEFs induce EGFR clustering and cause receptor aggregation independent of EGF treatment. **(B) MCF10CA1a:** iEFs have no effect of EGFR distribution, however, iEF treatment in presence of EGF results in downregulation of EGFR expression. **(C) MCF10A:** iEFs have no effect on EGFR aggregation/clustering. This figure has just the EGFR receptor channel (GFP channel from Fig 4 in the main text).

**
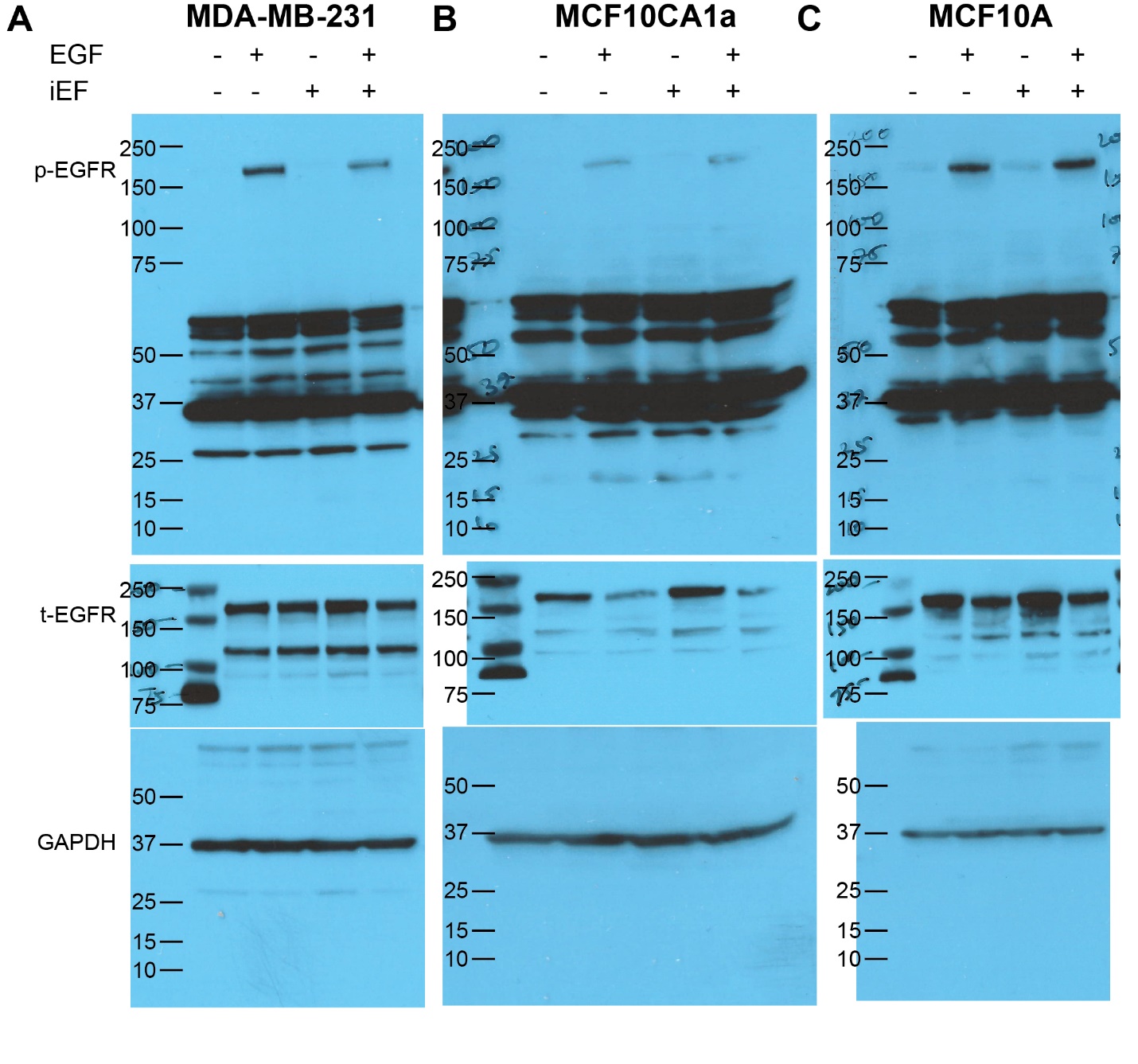
**

**Supplementary Figure 6. Raw western blot membranes for p-EGFR, t-EGFR, and GAPDH. (A) MDA-MB-231 cells. (B) MCF10CA1a cells. (C) MCF10A cells.**


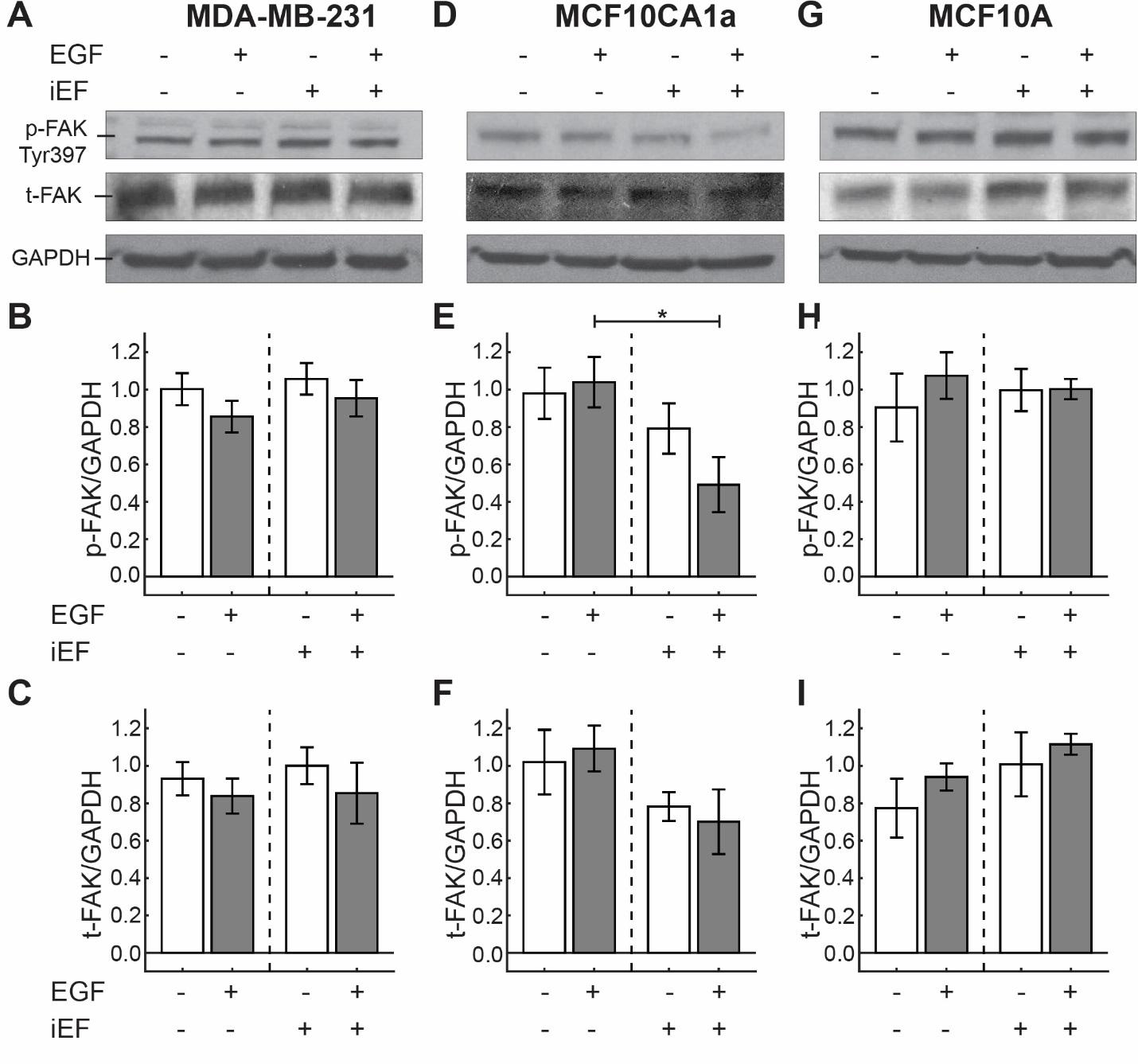


**Supplementary Figure 7. Effect of iEFs on levels of p-FAK and t-FAK levels. MDA-MB-231: (A)** Western blot analysis to examine the expression of p-FAK (Tyr397) and total FAK protein levels on treatment with EGF and iEFs. **(B)** Densitometry analysis of p-FAK (Tyr397) levels for the blots shown in (A). **(C)** Densitometry analysis of total FAK levels for the blots shown in (A). **MCF10CA1a: (D)** Western blot analysis to examine the expression of p-FAK (Tyr397) and total FAK protein levels on treatment with EGF and iEFs. **(E)** Densitometry analysis of p-FAK (Tyr397) levels for the blots shown in (D). **(F)** Densitometry analysis of total FAK levels for the blots shown in (D). **MCF10A: (G)** Western blot analysis to examine the expression of p-FAK (Tyr397) and total FAK protein expression levels on treatment with EGF and iEFs. **(H)** Densitometry analysis of p-FAK (Tyr397) levels for the blots shown in (G). **(I)** Densitometry analysis of total FAK levels for the blots shown in (G). All data presented as mean ± SE*_M_* (Pair-wise Student t-test, *p<0.05, **p<0.01, ***p<0.005, and ****p<0.001, N = 3 for each condition).


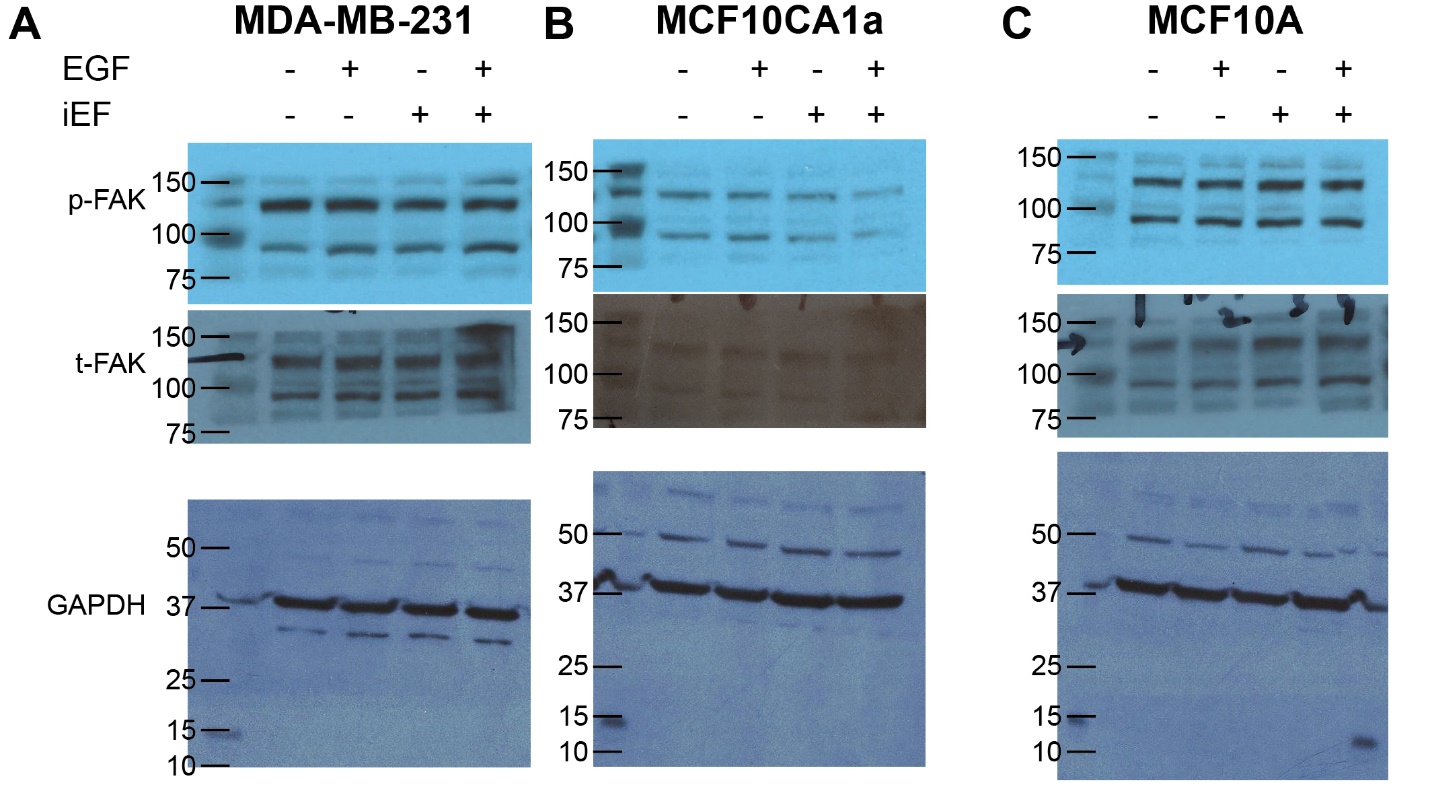


**Supplementary Figure 8. Raw western blot membranes for p-FAK, t-FAK, and GAPDH. (A) MDA-MB-231 cells. (B) MCF10CA1a cells. (C) MCF10A cells.**


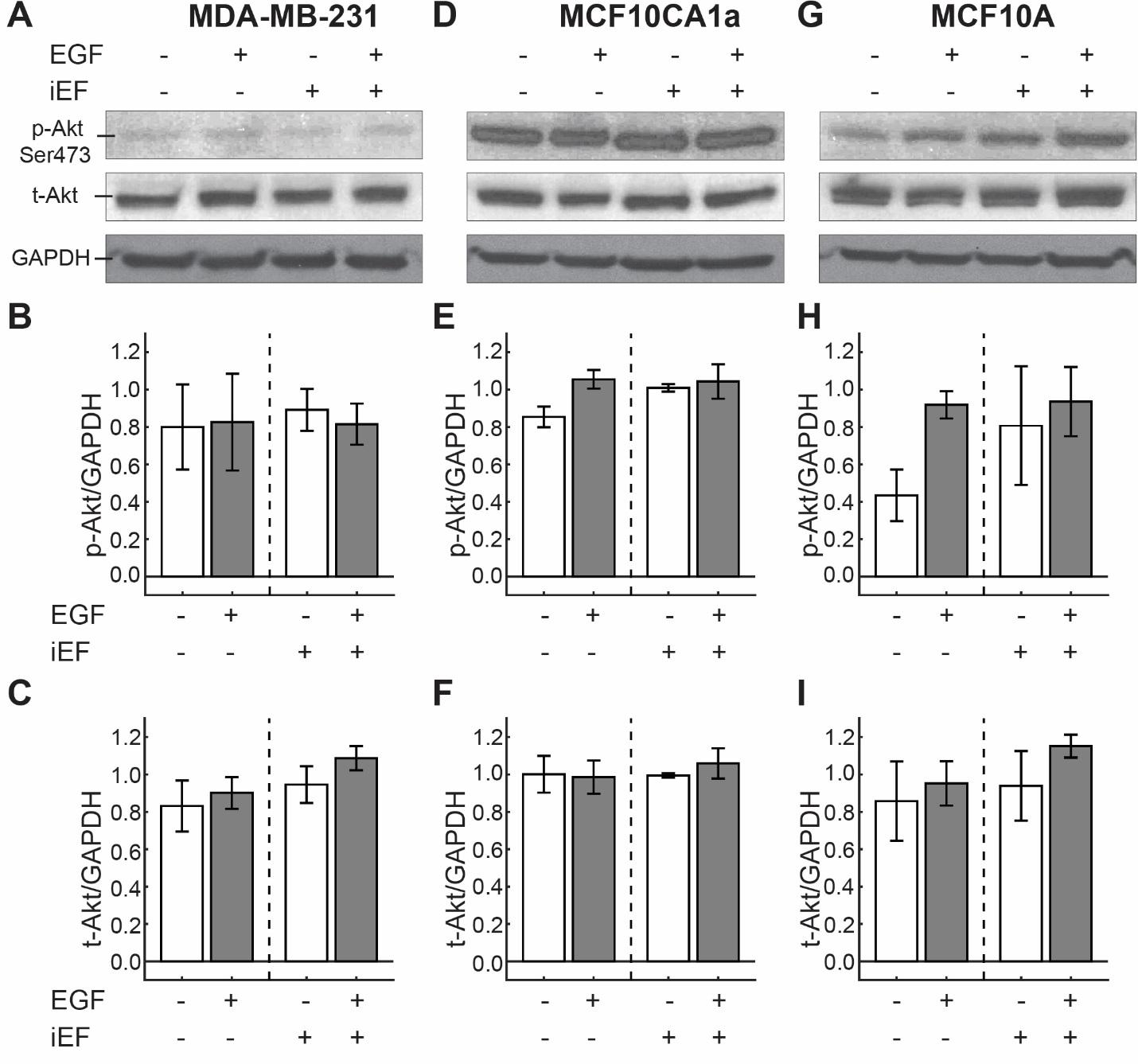


**Supplementary Figure 9. Effect of iEFs on levels of p-Akt and t-Akt levels. MDA-MB-231: (A)** Western blot analysis to examine the expression of p-Akt (Ser473) and total Akt protein levels on treatment with EGF and iEFs. **(B)** Densitometry analysis of p-Akt (Ser-473) levels for the blots shown in (A). **(C)** Densitometry analysis of total Akt levels for the blots shown in (A). **MCF10CA1a: (D)** Western blot analysis to examine the expression of p-Akt (Ser473) and total Akt protein levels on treatment with EGF and iEFs. **(E)** Densitometry analysis of p-Akt (Ser-473) levels for the blots shown in (D). **(F)** Densitometry analysis of total Akt levels for the blots shown in (D). **MCF10A: (G)** Western blot analysis to examine the expression of p-Akt (Ser473) and total Akt protein expression levels on treatment with EGF and iEFs. **(H)** Densitometry analysis of p-Akt (Ser-473) levels for the blots shown in (G). **(I)** Densitometry analysis of total Akt levels for the blots shown in (G). All data presented as mean ± SE*_M_* (Pair-wise Student t-test, *p<0.05, **p<0.01, ***p<0.005, and ****p<0.001, N = 3 for each condition).


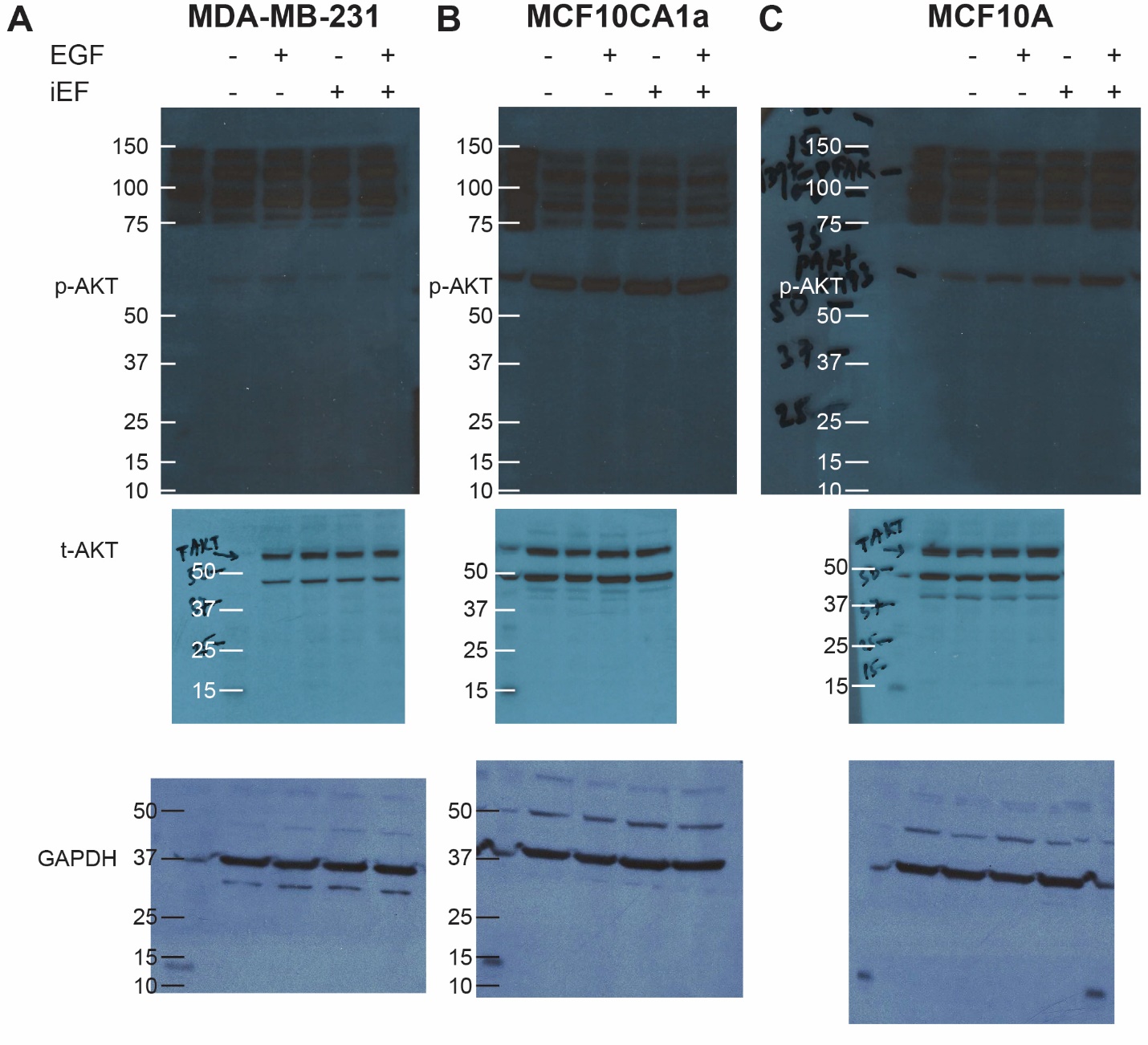


**Supplementary Figure 10. Raw western blot membranes for p-AKT, t-AKT, and GAPDH. (A) MDA-MB-231 cells. (B) MCF10CA1a cells. (C) MCF10A cells.**


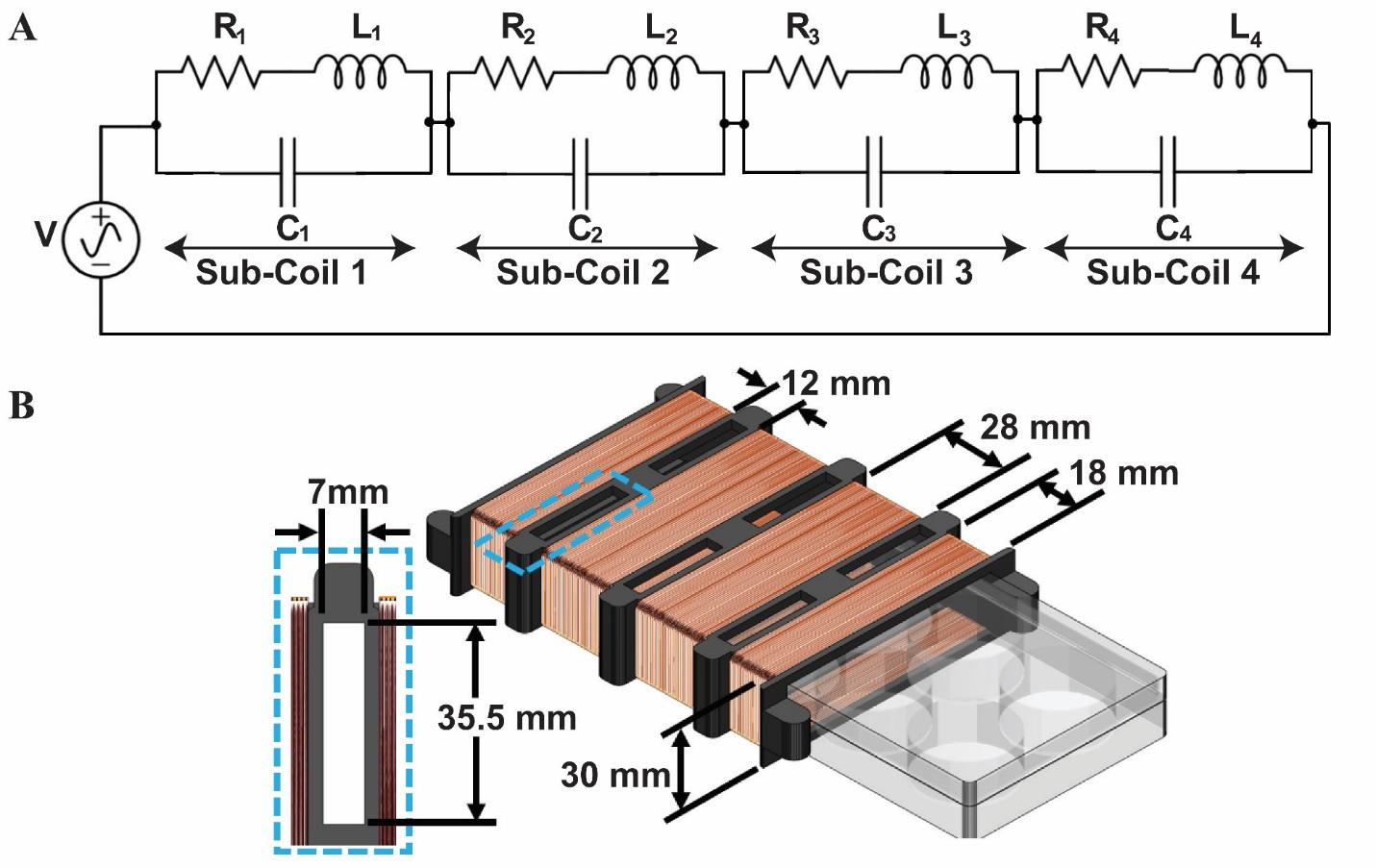


**Supplementary Figure 11. (A) Circuit Element Model.** Simplified circuit element model of Helmholtz coil used in experiments. The coil is composed of four similar coil segments in series. The first and fourth segments are identical, as are the second and third segments. **(B) Helmholtz Coil.** Schematics indicating the dimensions of the coil and the microscope viewing windows.


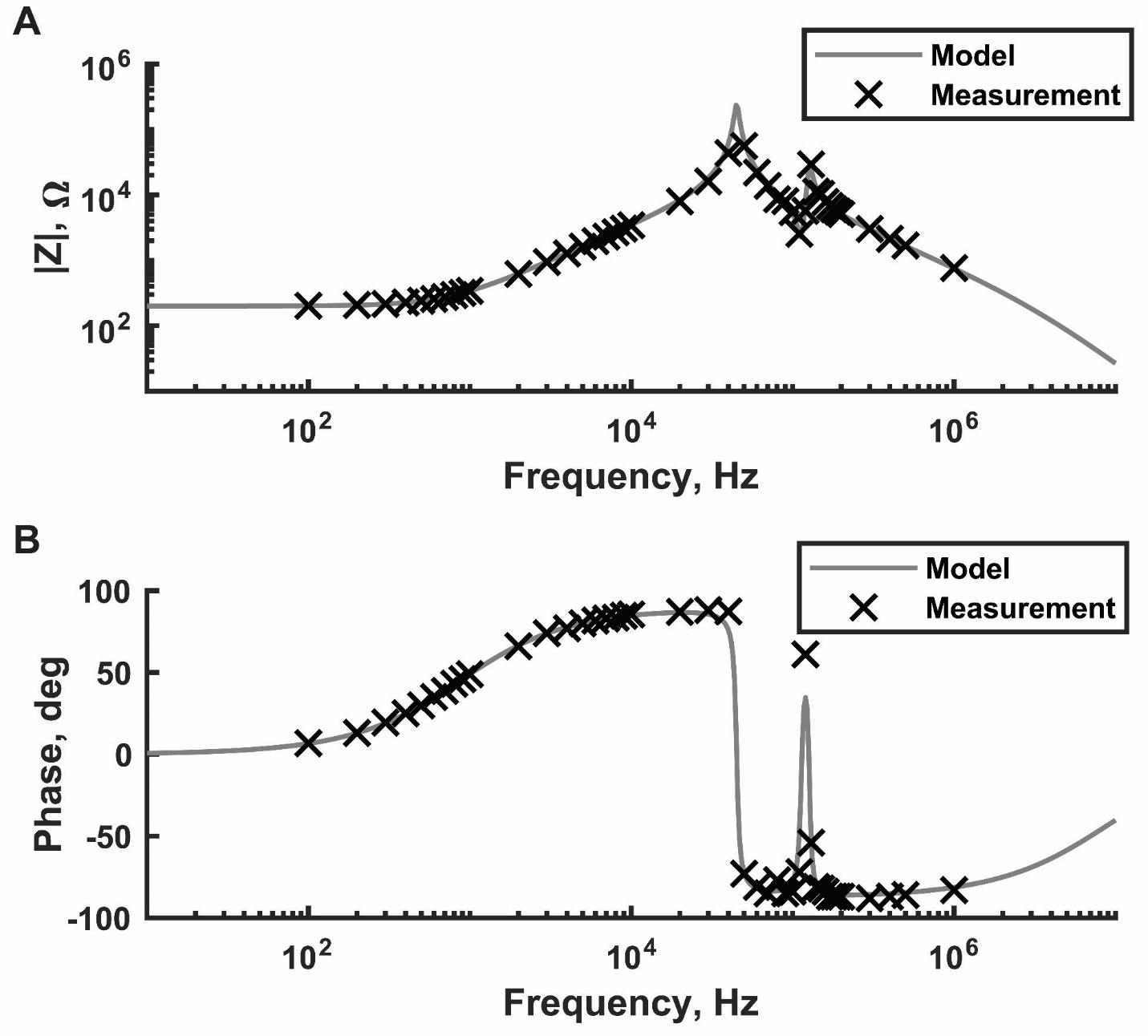


**Supplementary Figure 12. Frequency Response of Helmholtz Coil. (A) Impedance Measurement.** Frequency response of Helmholtz coil using simple circuit element model compared with measured values of impedance. **(B) Phase.** Frequency response of Helmholtz coil using simple circuit model compared with measured values of impedance phase for varying frequency inputs. The two resonant peaks correspond to inner and outer segments of the coil.


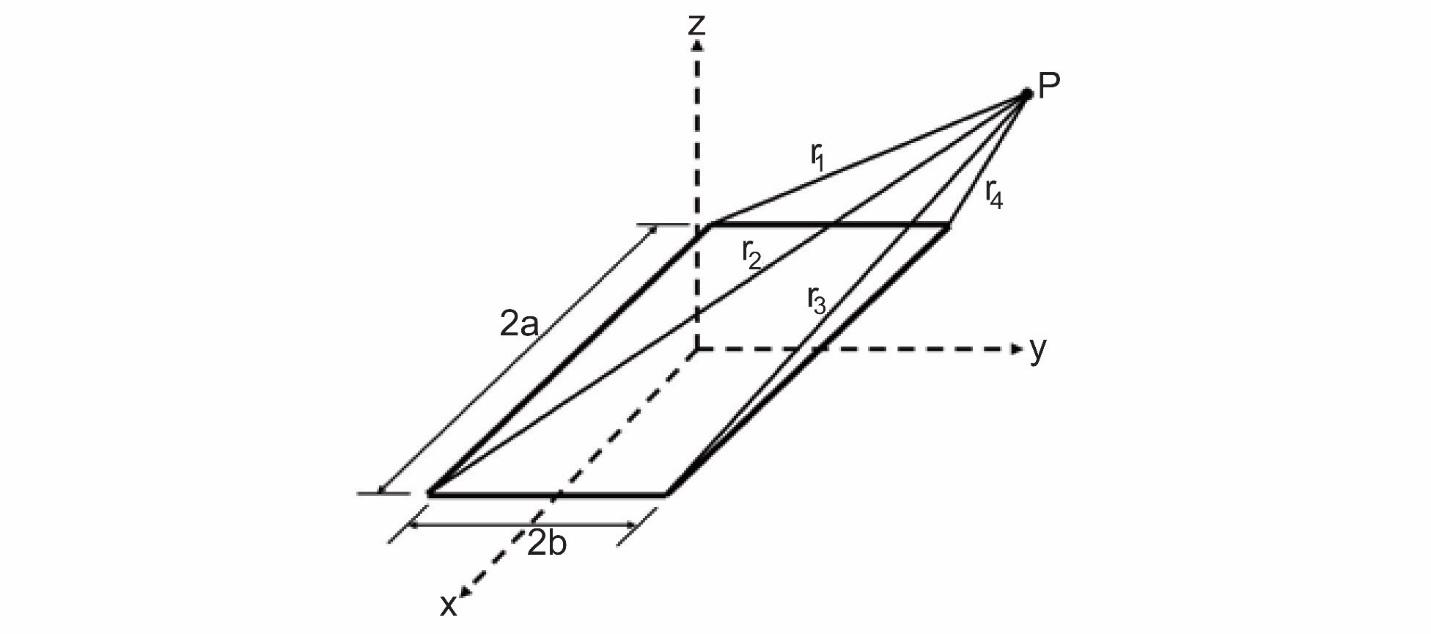


**Supplementary Figure 13.** Geometry of a single rectangular loop of current used for calculating the vector potential $\vec{A}$ at point P(x,y,z) with respect to a coordinate system.


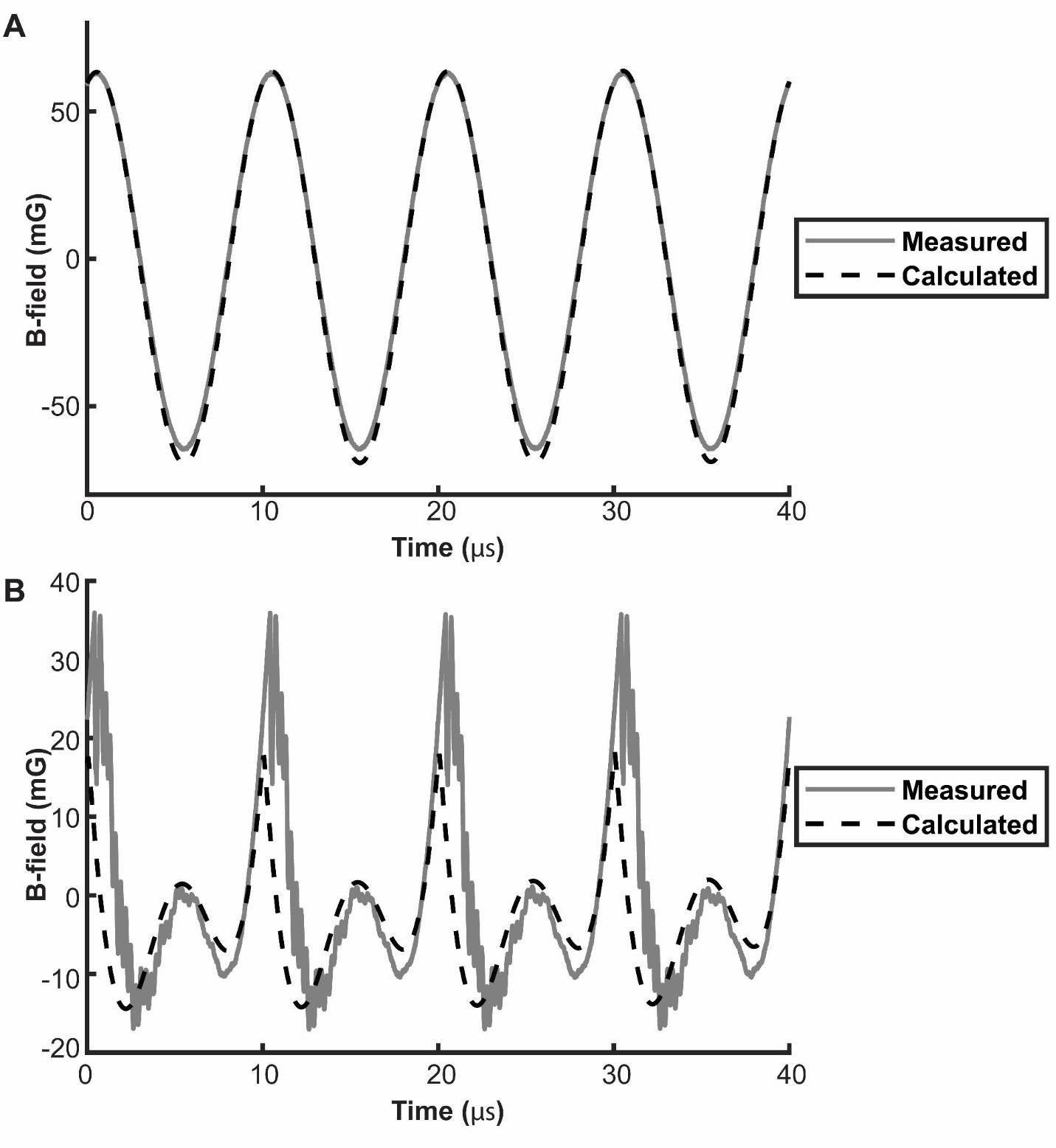


**Supplementary Figure 14. Comparison of measured and calculated B-field versus time.** **(A)** 100 kHz, 20 V_pp_ sinewave was applied to the coil using a function generator. The magnetic field probe was located at the center of the coil. **(B)** 100 kHz, 20 V_pp_ sawtooth waveform (Fig S1D) was applied to the coil using a function generator. The magnetic field probe was located at the center of the coil.

**Supplementary Discussion**

**Effect of iEFs on FAK activation and expression**

Focal adhesion kinase (FAK), a non-receptor protein kinase, is a potent driver of both tumor growth and metastasis, and is commonly overexpressed in invasive breast carcinoma^33^. FAK associated proteins have been shown to bind to actin and FAK is known to associate with Rho-GTPases, indicating that changes in FAK activation and/or expression can lead to changes in actin dynamics^34, 35^. Phosphorylation of FAK at Tyr397 and actin cytoskeleton integrity has previously been shown to be necessary for cell motility^36^. Therefore, we used Western blot analysis to determine if iEF treatment was changing activation and/or expression of FAK that could then be linked to changes observed in actin distribution and cell motility upon treatment with iEFs. We found that iEFs had no effect on the phosphorylation or total levels of FAK in MDA-MB-231 cells (**Supplementary** **Fig. 7A, B, C and 8**). In contrast, iEF treatment on EGF-treated MCF10CA1a breast cancer cells, resulted in significant downregulation of FAK phosphorylation (**Supplementary Fig. 7D, E, F and 8**) compared to EGF-treated cells in the absence of iEFs. Finally, iEFs had no effect on FAK activation or expression (**Supplementary Fig. 7G, H, I and 8**) for normal epithelial MCF10A cells. In summary, these results clearly illustrate the fact that while the functional responses to iEF treatment in different metastatic breast cancer cell lines may be similar, the signaling controlling these responses is cell line dependent. Clearly, changes observed in cell migration speeds and actin distribution were independent of FAK in MDA-MB-231 cells but similar changes in speed and actin distribution in MCF10CA1a cancer cells were in part regulated by changes in FAK activation.

**Supplementary Notes (custom codes)**

**Transwell Cell Counting Code**

close all

clear

clc

path = 'insert path';

cd(path);

% Grab image files

List = dir('*.tif');

% Total = zeros(1,6);

for i = 1:12

close all

% Grab image i

I = imread(List(i).name);

% Display image

imshow(I,'InitialMagnification',30)

% Radius of transwell membrane

radius = 1810/2;

% Select center of transwell

center = ginput(1);

% Grab size of image

[ny, nx, nz] = size(I);

% Create x and y arrays

x = 1:1:nx;

y = 1:1:ny;

% Generate position matrix

[X, Y] = meshgrid(x,y);

% Create mask of inner matrix

ind = (X-center(1)).^2+(Y-center(2)).^2<radius^2;

ind = uint8(double(ind));

% Split image into RGB components

Ir = I(:,:,1).*ind;

Ig = I(:,:,2).*ind;

Ib = I(:,:,3).*ind;

I = cat(3,Ir,Ig,Ib);

Im = rgb2gray(I);

% Reverse image

I = imcomplement(I);

Im = imcomplement(Im);

% Threshold using Otsu's method

[counts, ind] = imhist(I,64);

[T, EM] = otsuthresh(counts(1:end-1));

BW = imbinarize(Im,T*.75);

se = strel('disk',3);

% Open image

BW = imopen(BW,se);

% Identify objects

cc = bwconncomp(BW);

% Count objects

N = cc.NumObjects;

% Create list of object areas

celldata = regionprops(cc,'basic');

areas = [celldata.Area];

areas = sort(areas);

avg = geomean(areas(1:end-1));

count(i) = N;

for k = 1:length(areas)-1

if areas(k)/avg < 2

extra = 0;

else

extra = floor(areas(k)/avg)-1;

end

count(i) = count(i) + extra;

end

fprintf(['\n' List(i).name ' = %4.0f\n'],count(i));

fprintf('*******************************************************\n')

end

**Actin Polarization Code**

%%-----------Loading Image(s) and Some Preprocessing---------------------%%

clc

clear

close all

% Read Image - Actin (Only for MCF10IV)

img = imread('2.png');

% Display Image

figure(1), imshow(img,'InitialMagnification',60)

% Splitting Channels (Only for MDA-MB-231 or MCF10A)

a = zeros(size(img, 1), size(img, 2));

%red channel

red = img(:,:,1);

just_red = cat(3, red, a, a);

%green channel

green = img(:,:,2);

just_green = cat(3, a, green, a);

%blue channel

blue = img(:,:,3);

just_blue = cat(3, a, a, blue);

% converting to grey scale image

just_grey = rgb2gray(just_red);

blue_grey = rgb2gray(just_blue);

% Image Sharpening Step

% sharpimg8 = imsharpen(img_actin8); % Only for MCF10IV

sharpimg8 = imsharpen(just_grey); % Only for MDA-231/MCF10A

% Image filtering for Initial Background Removal - Median Filter

% filterimg8 = medfilt2(sharpimg8); % Only for MCF10IV

filterimg8 = medfilt2(sharpimg8); % Only for MDA-231/MCF10A

just_grey = filterimg8;

% blue_grey = img_dapi8;

%just_grey = imadjust(just_grey);

level = 0.05;

Ibw = im2bw(just_grey,level);

%%

%%----------------Selecting Custom ROI in an Image-----------------------%%

roi = imrect();

pos = wait(roi);

% Number of bins, can only be factor of 360

Num_Bins = 360;

bins1 = zeros(1,Num_Bins);

for Z = 1:1

cropImg = imcrop(just_grey, [pos(1)-Z*5 pos(2)-Z*5 pos(3)+2*Z*5 pos(4)+2*Z*5]);

cropImg_Ibw = imcrop(Ibw, [pos(1)-Z*5 pos(2)-Z*5 pos(3)+2*Z*5 pos(4)+2*Z*5]);

cropImg2 = imcrop(blue_grey, [pos(1)-Z*5 pos(2)-Z*5 pos(3)+2*Z*5 pos(4)+2*Z*5]);

%figure, imshow(cropImg), title('User Defined ROI')

%Background Subtraction

background = imopen(cropImg, strel('disk', 100));

background2 = imopen(cropImg2, strel('disk', 100));

i3 = cropImg-background;

i32 = cropImg2-background2;

%Contrast Enhancement

i4 = i3;

i5= imadjust(i3);

i42 = i32;

%%

%%-----------------------Centroid Calculation------------------------%%

level = graythresh(i4);

Ibw = cropImg_Ibw;

Ibw = imfill(Ibw,'holes');

Ibw = bwareaopen(Ibw,10);

Ibw = bwmorph(Ibw,'spur');

level2 = graythresh(i42);

Ibw2 = im2bw(i42,level2);

labels2 = logical(Ibw2);

labels = logical(Ibw);

stat=regionprops(labels,'Orientation','centroid','area','MajorAxisLength','MinorAxisLength');

CenX = 0;

CenY = 0;

for i = 1:length(stat)

Areas(i) = stat(i).Area;

CenX = CenX + stat(i).Centroid(1);

CenY = CenY + stat(i).Centroid(2);

end

[~, ind] = max(Areas);

orient=stat(ind).Orientation;

angle=0;

stat2 = regionprops(labels2,'area','centroid');

Large_Length = max([stat(ind).MajorAxisLength,stat(ind).MinorAxisLength]);

Small_Length = min([stat(ind).MajorAxisLength,stat(ind).MinorAxisLength]);

Avg_Length = (Large_Length+Small_Length)/2;

% Factor that determines how much distance from centroid weighs into answer

% Smaller => distance doesnt matter

Weight_Factor = Small_Length/Large_Length

% Centroid of Cell

CenX = CenX/length(stat);

CenY = CenY/length(stat);

%%

%%---------------------------Actin Distribution-------------------------%%

[m,n] = size(i4);

theta = zeros(m,n);

dist = zeros(m,n);

dist_max = zeros(1,Num_Bins);

bins = zeros(1,Num_Bins);

bins_pix = zeros(1,Num_Bins);

for j = 1:m

for k = 1:n

d = ((j-CenY)^2 + (k-CenX)^2);

x = j - CenY;

y = k - CenX;

phi = atand(x/y);

if(x<0 && y>0)

phi = abs(phi);

elseif (x<0 && y<0)

phi = 180 - abs(phi);

elseif (x>0 && y<0)

phi = 180 + abs(phi);

elseif (x>0 && y>0)

phi = 360 - abs(phi);

end

if (phi == 0)

phi = 360;

end

dist(j,k) = d;

theta(j,k) = ceil(phi*(Num_Bins/360));

end

end

for j = 1:m

for k = 1:n

value = double(i4(j,k))/Ibw(j,k);

if isnan(value) || isinf(value)

%do nothing

else

bins(1,theta(j,k)) = bins(1,theta(j,k)) + dist(j,k)^(Weight_Factor)*double(i4(j,k));

if (dist_max(1,theta(j,k)) < dist(j,k))

dist_max(1,theta(j,k)) = dist(j,k);

end

bins_pix(theta(j,k)) = bins_pix(theta(j,k)) + 1;

end

end

end

for j = 1:Num_Bins

if(bins_pix(1,j) == 0 || dist_max(1,j) == 0)

bins(1,j) = 0;

else

bins(1,j) = bins(1,j)/dist_max(1,j)^(Weight_Factor);

bins(1,j) = bins(1,j)/bins_pix(1,j);

end

end

for y=1:Num_Bins

z=y-ceil(angle*(Num_Bins/360));

if z<=0

z=z+Num_Bins;

end

if Z ==1

bins1(1,y) = bins(1,z);

else

bins1(1,y)= mean([bins1(1,y) bins(1,z)]);

end

end

end

beta = 1:1:Num_Bins;

alpha = 0:0.001:2*pi;

xunit = cos(alpha);

yunit = sin(alpha);

bins1(1,:) = smooth(bins1(1,:));

big = max(bins1(1,:));

bins1(1,:) = bins1(1,:)/big*max([m n]);

figure(6)

h = polar(0,max([m n]));

hold on

plot(xunit*max([m n]),yunit*max([m n]), 'LineWidth',2)

for i = 1:Num_Bins

xp(i,:) = [0 bins1(1,i)*cosd(i/(Num_Bins/360))];

yp(i,:) = [0 bins1(1,i)*sind(i/(Num_Bins/360))];

hold on

val = bins1(1,i)/max(bins1(1,:));

if val >= 0 && val < 0.2

plot(xp(i,:),yp(i,:),'LineWidth',2,'Color',[0 0 1*(1-val)])

elseif val >= 0.2 && val < 0.4

plot(xp(i,:),yp(i,:),'LineWidth',2,'Color',[0 1*(val) 1*(val)])

elseif val >= 0.4 && val < 0.6

plot(xp(i,:),yp(i,:),'LineWidth',2,'Color',[0 1*(val) 0])

elseif val >= 0.6 && val <0.8

plot(xp(i,:),yp(i,:),'LineWidth',2,'Color',[1*(val) 1*(val) 0])

else

plot(xp(i,:),yp(i,:),'LineWidth',2,'Color',[1*(val) 0 0])

end

end

axis square

hsp1 = get(gca, 'Position');

set(gca,'visible','off')

figure(5)

subplot(1,3,2)

image(i4)

axis equal

colormap(gca,jet)

set(gca,'visible','off')

subplot(1,3,1)

imshow(cropImg), hold on

plot(CenX,CenY,'ro');

axis equal

set(gca,'visible','off')

hi_actin = find(bins1 >= 0.8*max([m n]))/(Num_Bins/360);

p = max(size(hi_actin));

PR = 0;

for i = 1:p

if((hi_actin(i) >=70 && hi_actin(i)<=110) || (hi_actin(i) >=250 && hi_actin(i)<=290))

PR = PR + 1;

end

end

PR

p

PR = PR/p;

TAR=PR

ang = 0;

for i = 1:p

if(hi_actin(i) > 90 && hi_actin(i) <=180)

hi_actin(i) = 180 - hi_actin(i);

elseif (hi_actin(i) > 180 && hi_actin(i) <= 270)

hi_actin(i) = hi_actin(i) - 180;

elseif (hi_actin(i) > 270 && hi_actin(i) <=360)

hi_actin(i) = 360 - hi_actin(i);

end

end

AvgAngle=sum(hi_actin)/max(size(hi_actin))

**Supplementary Methods**

**Characterization of electric fields generated by the Helmholtz coil**

The Helmholtz coil used in some of the experiments reported in this paper comprises 4 segments. Supplementary Figure 11 shows a simplified circuit model of the Helmholtz coil showing these 4 segments, each comprising a resistance, capacitance, and inductance. The frequency response of the Helmholtz coil was determined by measuring the voltage drop across a 100 Ω sense resistor connected in series with the coil. A sine wave voltage waveform was then applied and the amplitude and phase of the signal across the resistor monitored. The impedance was inferred based on the applied potential and measured current through the resistor. The resonant frequencies of the inner and outer coil segments of the Helmholtz coil were determined from the two impedance peaks in the frequency response. Lump inductance and resistance were measured using an LCR meter (Keysight U1733C). Values of inductance and resistance of each coil segment were adjusted assuming the total inductance and resistance of the coil is an aggregate of the individual inductances and resistances of each coil segment. To match the resonant frequency peaks, the capacitance of each coil segment was determined by the inferred inductance based on the relation: $f=\frac{1}{(2\pi\sqrt{LC})}$. Supplementary Fig. 12 shows a comparison of the circuit element model using the fit parameters with measured values of the frequency response of the coil.

Closed form solutions for the vector potential **A** and magnetic induction **B** for a Helmholtz coil of rectangular cross section, are available in the literature^1^. The depiction of the various quantities appearing in the formulae given below is given in Supplementary Fig. 13. The relevant expressions for the components of the vector potential **A,** induced electric field **E,** and magnetic induction **B** at a point *P*(*x*, *y*, *z*) are:

$A_{x}=\frac{\mu_{0}I}{4\pi}ln \left[ \frac{{(r}_{1}+a+x)}{(r_{2}-a+x)}\frac{(r_{3}-a+x)}{(r_{4}+a+x)} \right]$ (S1)

$A_{y}=\frac{\mu_{0}I}{4\pi}ln \left[ \frac{{(r}_{2}+b+y)}{(r_{3}-b+y)}\frac{(r_{4}-b+y)}{(r_{1}+b+y)} \right]$ (S2)

$\vec{\mathbf{E}}=-\frac{\partial\vec{\mathbf{A}}}{\partial t}$ (S3)

$E_{x}=-\frac{dI}{dt}\frac{\mu_{0}}{4\pi}ln \left[ \frac{{(r}_{1}+a+x)}{(r_{2}-a+x)}\frac{(r_{3}-a+x)}{(r_{4}+a+x)} \right]$ (S4)

$E_{y}=-\frac{dI}{dt}\frac{\mu_{0}}{4\pi}ln \left[ \frac{{(r}_{2}+b+y)}{(r_{3}-b+y)}\frac{(r_{4}-b+y)}{(r_{1}+b+y)} \right]$ (S5)

$B_{z}=\frac{\mu_{0}I}{4\pi}\sum_{n=1}^{4} \left[ \frac{\left( -1 \right)^{n}d_{n}}{r_{n}\left[ r_{n}+\left( -1 \right)^{n+1}C_{n} \right]}-\frac{C_{n}}{r_{n}\left[ r_{n}+d_{n} \right]} \right]$ (S6)

where:

$C_{1}=-C_{4}=a+x$

$C_{2}=-C_{3}=a-x$

$d_{1}=d_{2}=y+b$

$d_{3}=d_{4}=y-b$

$r_{1}=\sqrt{\left( a+x \right)^{2}+\left( y+b \right)^{2}+z^{2}}$

$r_{2}=\sqrt{\left( a-x \right)^{2}+\left( y+b \right)^{2}+z^{2}}$

$r_{3}=\sqrt{\left( a-x \right)^{2}+\left( y-b \right)^{2}+z^{2}}$

$r_{4}=\sqrt{\left( a+x \right)^{2}+\left( y-b \right)^{2}+z^{2}}$

Each winding is treated as being an ideal loop of current and electric field and magnetic induction are calculated assuming that the respective contributions of each winding can be superposed at a specific location in space. The current through each loop was determined based on the current through the inductive branch of the circuit model shown in Supplementary Fig. 11. A comparison of the calculated magnetic field versus the measured magnetic field (using a magnetic field probe, Magnetic Sciences, Model #MC162) for a sine wave is shown in Supplementary Fig. 14A for the center position of the coil. Supplementary Fig. 14B shows a comparison of the measured and calculated field for a sawtooth waveform (**Supplementary Fig. 1D**). Although the measurement shows high frequency artifacts picked up by the probe, the model predicts that no such high frequency fields should exist so that these artifacts are likely related to the measurement. The discrepancy may be a result of capacitive coupling of the sensor with the probe during the measurement.

**Characterization of chemokine gradients in the MBDM assay**

For the detailed characterization of chemokine gradients in the MBDM assay, we adopted a three-pronged approach. We first estimated the diffusion coefficient using the Stokes-Einstein equation, with the inherent assumption that the molecules of the fluorescently conjugated dye are spherical with a hydration radius of 2.3 nm for 10 kDa dextran^2^. The diffusivity as determined by the Stokes-Einstein equation is given by:

$D_{0}=\frac{k_{B}T}{6\piɳR_{H}}$ (S7)

where *D_0_* is the diffusivity in m^2^s^-1^, *k_B_* is Boltzmann’s constant (1.3806 x 10^-23^ JK^-1^), *T* is the temperature in K, *η* is the viscosity in N-sm^-2^, and *R_H_* is the radius of the hydrated molecule. At 298 K, for *η* = 8.9 x 10^-4^ N-sm^-2^ and *R_H_* = 2.3 nm, the value of *D_0_* given by this formula is 106.6 µm^2^s^-1^ (1.066 x 10^-10^ m^2^s^-1^).

The profiles of chemokine gradients were calculated using the Stokes-Einstein diffusivity by solving the one-dimensional, transient, diffusion equation and compared with experimental measurements of the chemokine gradient profiles (**Supplementary** **Fig 2)** over 12 hours using a 10 kDa FITC-conjugated dextran dye. The 1-D, transient diffusion equation is given by:

$\frac{\partial C}{\partial t}= D_{0}\frac{\partial^{2}C}{\partial x^{2}}$ (S8)

where *C* is the concentration of the dye, *t* is time, *x* is distance along the microchannel, and *D_0_* is the diffusivity. Assuming the intensity of the fluorescing dye, *I*, is proportional to its concentration, Equation S7 can be re-written in terms of intensity:

$\frac{\partial I}{\partial t}= D_{0}\frac{\partial^{2}I}{\partial x^{2}}$ (S9)

Equation S8 was solved numerically using COMSOL Multiphysics 5.3a, to calculate the gradient profiles over a 12-hour period. The diffusivity *D_0_* was varied parametrically from 10^-7^ to 10^-14^ m^2^s^-1^, and the calculated profiles of *I(x, t)* were compared to measurements. A value of *D* = 1.74 x 10^-10^ ± 9.33 x 10^-11^ m^2^s^-1^ was determined by comparison between calculations and experimental measurement with an r-squared > 0.9. It can be seen that the experimentally determined diffusivity is on the same order as the theoretical value calculated using the Stokes-Einstein equation (1.066 x 10^-10^ m^2^s^-1^).

Based on the measured and calculated gradient profiles, we can be confident that for the duration of our migration experiments, the MBDM Assay is able to sustain stable gradients ensuring a chemotactic migratory response instead of a chemokinetic migratory response.

**Calculation of cell mean speed and persistence in the MBDM assay**

To calculate persistence, the following protocol was followed for each cell tracked in a microtrack of the MBDM assay (**Supplementary** **Fig S3**). A cell starts at position *P_t_* at time *t*. After a single time increment, *dt*, the cell moves to a new position, *P_t + dt_*. The distance traveled during this time is the absolute difference in the two positions denoted as *L_i_* :

$L_{i}=\left| P_{t+dt}-P_{t} \right|$

The total distance traveled is then the sum of all distances traveled during *N* time increments denoted as *L_t_*. Additionally, the total time of travel is the number of time increments multiplied by the size of the time increments denoted as *T_t_*.

$L_{t}=\sum_{i=1}^{N} L_{i}$

$T_{t}=N\cdot dt$

The displacement is simply the difference between the final and initial position:

$L_{d}=\left| P_{Final}-P_{Initial} \right|$

Therefore the mean speed can be calculated as follows:

$Mean Speed=\frac{L_{t}}{T_{t}}$

The persistence can be calculated as follows:

$Persistence=\frac{L_{d}}{L_{t}}$

**SUPPLEMENTARY REFERENCES**

1. Misakian, M., Equations for the magnetic field produced by one or more rectangular loops of wire in the same plane. Journal of research of the National Institute of Standards and Technology, 2000. 105(4): p. 557.

2. Watson, P.M.D., et al., Modelling the endothelial blood-CNS barriers: a method for the production of robust in vitro models of the rat blood-brain barrier and blood-spinal cord barrier. BMC neuroscience, 2013. 14(1): p. 59.

3. Sulzmaier FJ, Jean C, & Schlaepfer DD (2014) FAK in cancer: mechanistic findings and clinical applications. *Nature reviews Cancer* 14(9):598.

4. Mitra SK, Hanson DA, & Schlaepfer DD (2005) Focal adhesion kinase: in command and control of cell motility. *Nature reviews Molecular cell biology* 6(1):56.

5. Parsons JT, Horwitz AR, & Schwartz MA (2010) Cell adhesion: integrating cytoskeletal dynamics and cellular tension. *Nature reviews Molecular cell biology* 11(9):633.

6. Sieg DJ*, et al.* (2000) FAK integrates growth-factor and integrin signals to promote cell migration. *Nature cell biology* 2(5):249.
