# Supplementary material for: Electromagnetic fields alter the motility of metastatic breast cancer cells
Source: Commun Biol. 2019 Aug 8;2:303. doi: 10.1038/s42003-019-0550-z (PMC6687738; doi:10.1038/s42003-019-0550-z)
Supplement: Supplementary file 2 — Description of Additional Supplementary Files [file 42003_2019_550_MOESM2_ESM.pdf]

Supplementary Movie 1. Effect of iEFs on MDA-MB-231 cell motility in presence and absence of EGF gradients in the MBDM Assay. This movie shows single GFP-tagged MDA-MB-231 cells migrating from the cell-seeding port (bottom) to the media port (top). This illustrates the context specific dual role of iEFs both as a pro-migratory cue and an anti-migratory cue. The white dotted lines indicate the microtrack walls (20 µm in width) and the red line indicates the level of migration in case of controls (iEF-/EGF-). The duration of the movies is 12 hours and the images were taken in 5-minute intervals.

Supplementary Data 1. This is compilation of all the raw data that has been presented in form of graphs in the main text and the supplementary information. It contains the values for individual biological replicates for mean migration speeds, persistence, polarization ratio, migration numbers in Transwell Assay, and densitometry analysis of western blots for MDA-MB-231, MCF10CA1a, and MCF10A cells.
