# Supplementary material for: Electromagnetic fields alter the motility of metastatic breast cancer cells
Source: Commun Biol. 2019 Aug 8;2:303. doi: 10.1038/s42003-019-0550-z (PMC6687738; doi:10.1038/s42003-019-0550-z)
Supplement: Supplementary file 5 — Reporting Summary [file 42003_2019_550_MOESM5_ESM.pdf]

nature research

Corresponding author(s): Jonathan W. Song, Vish V. Subramaniam

Last updated by author(s): 07/09/2019

Reporting Summary

Nature Research wishes to improve the reproducibility of the work that we publish. This form provides structure for consistency and transparency in reporting. For further information on Nature Research policies, see Authors & Referees and the Editorial Policy Checklist.

Statistics

For all statistical analyses, confirm that the following items are present in the figure legend, table legend, main text, or Methods section.

n/a	Confirmed	
☐	☒	The exact sample size (*n*) for each experimental group/condition, given as a discrete number and unit of measurement
☐	☒	A statement on whether measurements were taken from distinct samples or whether the same sample was measured repeatedly
☐	☒	The statistical test(s) used AND whether they are one- or two-sided<br>*Only common tests should be described solely by name; describe more complex techniques in the Methods section.*
☒	☐	A description of all covariates tested
☐	☒	A description of any assumptions or corrections, such as tests of normality and adjustment for multiple comparisons
☐	☒	A full description of the statistical parameters including central tendency (e.g. means) or other basic estimates (e.g. regression coefficient) AND variation (e.g. standard deviation) or associated estimates of uncertainty (e.g. confidence intervals)
☐	☒	For null hypothesis testing, the test statistic (e.g. *F*, *t*, *r*) with confidence intervals, effect sizes, degrees of freedom and *P* value noted<br>*Give P values as exact values whenever suitable.*
☒	☐	For Bayesian analysis, information on the choice of priors and Markov chain Monte Carlo settings
☐	☒	For hierarchical and complex designs, identification of the appropriate level for tests and full reporting of outcomes
☒	☐	Estimates of effect sizes (e.g. Cohen's *d*, Pearson's *r*), indicating how they were calculated

Our web collection on statistics for biologists contains articles on many of the points above.

Software and code

Policy information about availability of computer code

Data collection	ImageJ with MTRACKJ Plugin was used for analysis of migration speeds and persistence<br>Custom MATLAB codes for calculation of numbers migrated (transwell assay), Polarization ratio (Actin quantification) were provided in supplementary information
Data analysis	All statistical analysis was done in JMP 14 (SAS) and IBM SPSS Statistics 25

For manuscripts utilizing custom algorithms or software that are central to the research but not yet described in published literature, software must be made available to editors/reviewers. We strongly encourage code deposition in a community repository (e.g. GitHub). See the Nature Research guidelines for submitting code & software for further information.

Data

Policy information about availability of data

All manuscripts must include a data availability statement. This statement should provide the following information, where applicable:

- Accession codes, unique identifiers, or web links for publicly available datasets
- A list of figures that have associated raw data
- A description of any restrictions on data availability

A data availability statement has been incorporated in the main text. Data will be readily available when requested from the corresponding author(s).

Field-specific reporting

Please select the one below that is the best fit for your research. If you are not sure, read the appropriate sections before making your selection.

☒ Life sciences ☐ Behavioural & social sciences ☐ Ecological, evolutionary & environmental sciences

For a reference copy of the document with all sections, see nature.com/documents/nr-reporting-summary-flat.pdf

Life sciences study design

All studies must disclose on these points even when the disclosure is negative.

Sample size	So every experiment was conducted three independent times (i.e. each experiment was setup on a different day considered as a biological replicate - repeated 3 times). Since all experiments involved single cell analysis, the number of cells analyzed varied from experiment to experiment. For experiments with just a single comparison between two conditions and where normality could not be tested, a two-tailed unpaired student t-test was used the p-value<0.05 considered as significant difference. However, wherever applicable, the normality of data was checked. In our case out data sets did not have a normal distribution therefore, we used independent samples Kruskal-Wallis Test (Non-parametic testing) to check if the the different conditions had different distributions. This was followed by post-hoc testing where the conditions were compared pair-wise. In this case, the significance values were adjusted by the Bonferroni Correction where the test stastic was also adjusted fro ties.
Data exclusions	For migrating cells, the cells that started dividing were excluded from the analysis. Also, cells that moved less than 1 cell length (<10 um) over the period of observation were excluded from the analysis.
Replication	Each experiment was conducted at least three independent times.
Randomization	Experiments were done in 6 well plates and devices were placed randomly between replicates therefore ensuring that any effect observed was not due to random variables such as fixed condition for fixed plate location. Moreover, this ensured that the on-stage incubator on the time-lapse microscopes did not result in changes observed as different conditions were randomized between wells between biological replicates. The direction of parallel and anti-parallel field with respect to Well 1 of the six well was changed between replicates to ensure complete randomization of conditions between replicates.
Blinding	Some of the data sets (migration speeds, persistence, and polarization ratio) analyzed by Jessica Ferree and Prabhat Kumar were given to them as blind sets and results provided by them were then compiled together to prevent human bias during analysis of experiments. However, the analysis done by Ayush Arpit Garg was not blind as the experiments were conducted by him so it was not possible to analyze these sets blindly.

Reporting for specific materials, systems and methods

We require information from authors about some types of materials, experimental systems and methods used in many studies. Here, indicate whether each material, system or method listed is relevant to your study. If you are not sure if a list item applies to your research, read the appropriate section before selecting a response.

Materials & experimental systems

n/a	Involved in the study	
☐	☒	Antibodies
☐	☒	Eukaryotic cell lines
☒	☐	Palaeontology
☒	☐	Animals and other organisms
☒	☐	Human research participants
☒	☐	Clinical data

Methods

n/a	Involved in the study	
☒	☐	ChIP-seq
☒	☐	Flow cytometry
☒	☐	MRI-based neuroimaging

Antibodies

Antibodies used	Alexa Fluoro® 488-conjugated Phalloidin - ThermoFisher - Cat#: A12379 - Lot#: 1583098, Alexa Fluoro® 555-conjugated Phalloidin - ThermoFisher - Cat#: 34055 - Lot#: 1853329, ActinRedTM 555 ReadyProbes® Reagent - ThermoFisher - Cat#: R37112 - Lot#: 1878888,EGFR Monoclonal Antibody (199.12) - ThermoFisher - Cat#: MA5-13319 - Lot #:SA2331448 , pEGFR - Cell Signaling Technology - Cat #: 3777S, pAkt - Cell Signaling Technology - Cat #: 9271S, pFAK - ThermoFisher - Cat #: 700255, t-EGFR - Santa Cruz - Cat#: SC-03-G, t-Akt - Santa Cruz - Cat#: SC-8312, t-FAK - Cell Signaling Technology - Cat #: 3285S, GAPDH - Cell Signaling Technology - Cat #: 5174S
Validation	Followed the manufacturer's protocols<br>For the the immunofluorescence of t-EGFR (invlovling primary and secondary anti-bodies) - negative control with just secondary antibodies without the primary antibody - this case did not generate any fluorescence signal, but as expected in presence of primary anti-body, the secondary antibody attached to the EGFR receptor which was then visible under the microscope

Eukaryotic cell lines

Policy information about cell lines

Cell line source(s)	MDA-MB-231 - Gary Luker Lab, University of Michigan, Ann Arbor,<br>MCF10CA1a - Ganju Lab, The Ohio State University, Columbus,<br>MCF10A - Ostrowski Lab, Medical University of South Carolina, Charleston (previously at The Ohio State University, Columbus)
Authentication	None of the cell lines were authenticated
Mycoplasma contamination	During immunoflourescence staining of the nucleus, the DAPI stain was found to the completely localized to the nucleus. In case of mycoplasma contamination, the cytoplasm lights up during imaging of the DAPI stain with the stain appearing as small blue dots dispersed throughout the cell cytoplasm. We never found this to the case in any of our cell lines. Therefore, we concluded that we did not have any mycoplasma contamination in our cell line cultures.
Commonly misidentified lines<br>(See ICLAC register)	None of the cell lines used in this study appear on the database of commonly misidentified lines. We confirmed this by cross-referencing the cell lines used in this study with the cells lines in the data base.
